# The Repeatability and Agreement of Ocular Parameters Measured with the MYAH and Myopia Master Devices between Expert and Non-Expert Practitioners

**DOI:** 10.3390/life14030407

**Published:** 2024-03-19

**Authors:** Sara Ortiz-Toquero, Irene Sanchez, Carmen Gurrea, Alba Recio, David Balsa, Raul Martin

**Affiliations:** Optometry Research Group, IOBA Eye Institute, Department of Theoretical Physics, Atomic and Optics, University of Valladolid, 47011 Valladolid, Spain

**Keywords:** axial length, myopia control, MYAH, Myopia Master, precision

## Abstract

In myopia control, it is essential to measure the axial length (AL) and corneal parameters, and to monitor whether changes in these parameters have occurred over time. The aim of this study was to analyse the repeatability and agreement between expert and non-expert practitioners in ocular parameters measured by the MYAH and Myopia Master. Three repeated measurements (*n* = 42) were recorded with the MYAH and Myopia Master by two (an expert and a non-expert) observers in a randomized order. The AL, K1, K2, and white-to-white (WTW) distance were collected. The intraobserver repeatability was excellent in all parameters measured with both devices in both observers. The AL outcome presented the best repeatability with the MYAH and Myopia Master (intraclass correlation coefficient, ICC = 1.0; coefficient of variation, CV ≤ 0.06% for both observers), while the WTW presented poorer results (ICC ≤ 0.991; CV ≤ 0.52%). The Myopia Master provides a significantly (*p* ≤ 0.01) flatter K1 and K2 as well as a lower WTW (*p* ≤ 0.01) than the MYAH. No statistically significant difference in AL measurements was found with either device (*p* ≥ 0.10; ICC = 1.0). None of the parameters showed differences (*p* ≥ 0.12) between the expert and non-expert observer. The MYAH and Myopia Master provide consistent measurements in a healthy adult population regardless of the previous clinical experience of the observer. AL measurements should be used interchangeably but K1, K2, and WTW should be used interchangeably with caution.

## 1. Introduction

Myopia is one of the major causes of severe visual loss; its incidence is increasing worldwide but is particularly marked in Asian countries [1,2]. It is estimated that by 2050, half of the population of the world will have myopia [1]. A complex interplay between genetic, ethnic, binocular vision (high levels of esophoria and high accommodation lag), and environmental factors (less time spent outdoors and prolonged indoor work tasks) play an important role in the onset and development of myopia [3]. When myopia progresses, the elongation of the eye increases, and when occur an excessive axial elongation, it can cause important ocular complications, such as retinal detachment, posterior vitreous detachment, maculopathy, glaucoma, or cataracts, with social and health consequences that also entail [4].

Currently, different methods are available to slow myopia progression among children and teenagers, such as the use of atropine [5], orthokeratology [6], peripheral defocus contact lens [7], or defocus incorporated multiple segment (DIMS) spectacles [8] that can reduce eyes’ axial length (AL) increase [9,10]. AL measured with partial coherence interferometry devices represents the interferometry between the surface of tear film and the retinal pigment epithelium [11] and is the main ocular parameter used in the clinic and research practice to monitor myopia progression [9,10]. Therefore, in a patient follow-up for myopia control, it is essential to measure the eyes’ AL and to monitor whether AL changes have occurred over time [12,13].

Due to the growing interest in myopia control treatments and the need to follow these patients closely, several biometric devices have been developed and launched on the market to be used for myopia control. This is the case for the multidiagnostic platform MYAH (Topcon Healthcare Inc., Tokyo, Japan) and the Oculus Myopia Master (Oculus GmBH, Wetzlar, Germany). Both devices, using different technologies, provide repeatable [13,14] measurements of AL; the MYAH device uses low coherence interferometry (830 nm diode laser) and the Myopia Master uses dual partial coherence interferometry. The relevant biometric eye parameters, such as flat (K1) and steep (K2) corneal curvature and white-to-white (WTW) distance, are provided by Placido rings disk analysis in the MYAH device and after the assessment of the reflections of a test spot and a central ring projected on the central 15 degrees of the cornea by the Myopia Master device. However, to ensure that myopia progression is being correctly monitored by analysing the ocular parameters, reliable measurements must be collected with these devices, but the acquisition procedure with both devices requires manual centration moving the device’s joystick in the necessary directions according to the alignment guides to collect data without self-centering tool, and no previous reports have described the impact of observer experience on the accuracy and agreement of measurements collected by these both devices.

For this reason, the purpose of this study was to assess the intraobserver repeatability and the interdevice and interobserver agreement between the expert and non-expert observer in AL, K1, K2, and WTW measurements performed by the MYAH and Myopia Master devices in a sample of healthy subjects.

## 2. Materials and Methods

### 2.1. Subjects

This study was designed as a prospective cross-sectional study. The Human Sciences Ethics Committee of Valladolid Area-Este Clinic Hospital (Castilla y Leon Public Health System-SACYL) approved this study, which followed the tenets of the Declaration of Helsinki. All participants were informed about this study and signed informed consent forms.

Healthy subjects with a best corrected visual acuity equal to or greater than 6/6 after a complete eye examination (medical history, visual acuity measured with Snellen charts, eye refraction (sphere, cylinder, axe), slit lamp, and fundus examination) were included in this study. Those with any active ocular surface disease, corneal opacities, glaucoma, use of medication that could affect the ocular physiology, or a history of any type of ocular surgery were excluded [15]. Before the eye examination, contact lens wear was discontinued for at least 2 days [16]. Only data from the right eye of each subject were chosen for the study analysis [17]. The mean spherical equivalent refractive was included as descriptive data for the sample and was calculated by adding the sum of the sphere power with half of the cylinder power.

### 2.2. Devices

The MYAH device is a multidiagnostic platform designed to evaluate the AL with optical low coherence interferometry (830 nm diode laser), analyse the corneal curvature with 24 Placido rings disk reflected from the anterior corneal curvature, and conduct pupillometry, incorporating a dry eye module [12,13]. This device performs six individual AL interferometric measurements per exam in the range of 15–38 mm and shows the mean values per measurement. Myopic progression is calculated with the Tideman curves integrated into the multidiagnostic platform software.

Myopia Master is a non-invasive biometer and autorefractometer device based on dual partial coherence interferometry. This device has an integrated keratometer that analyses the reflections of test spots and a central ring projected on the central 15 degrees of the cornea to measure the corneal curvature and the objective refraction values. Myopia Master performs six individual interferometric analyses per exam (880 nm infrared superluminescence diode) to provide the average AL measurement [13]. This device provides patient myopic progression curves over the years, comparing current measurements of each patient with a database of more than 20,000 eyes developed by the Brien Holden Vision Institute, which can be used to guide the eye care professional in the management of myopia.

### 2.3. Measurement Procedure

Two observers participated in this study: one expert observer (an optometrist with more than 10 years of clinical practice experience) and a non-expert observer (who was a final-year optometry degree student). Both observers had no previous experience in handling the MYAH or Myopia Master devices and received the same basic instructions and training about how to operate with both devices before commencing this study.

In a single visit, each observer performed three measurements [18,19] with both devices in all patients in a randomized order, following the guidelines provide by the manufacturer, in a darkened room after verification of instrument calibration. Examiners were only aware of their own results. The measurement procedure was carried out following the manufacturer’ recommendations in the instructions. Non-reliable measurements were discarded (the MYAH device provides an icon to discard non-reliable measurements, and the Myopia Master provides corneal measurements with a quality lower than 7), and only high-quality measurements were included in the statistical analysis. Randomization order was defined with a random number table created with Excel 2402 [Microsoft 365 Office Excel (Microsoft Corp., Redmond, WA, USA)]. The subject placed their chin on the chin rest, and the forehead was pressed against the forehead strap. The measurement was performed after an eye blink when the eye was aligned to the visual axis. The subjects moved their chin from the chin rest between scans to eliminate the interdependence of successive measurements. The AL, K1, K2, and WTW distances measured with both devices and both observers were collected.

### 2.4. Statistical Analyses

Statistical analyses were performed using SPSS for Windows 10 Pro software (version 26.0; SPSS, Inc., Chicago, IL, USA) and Microsoft Office Excel (Microsoft Corp., Redmond, WA, USA). The normality of data distributions was checked with the Kolmogorov–Smirnov test (*p* > 0.05 indicates that the data are normally distributed) [17,20]. Descriptive results are given as the mean, standard deviation (SD), and maximum and minimum range values; also, non-missing data and outliers were detected in the dataset.

This study followed the definition of intraobserver repeatability according to the British Standards Institute and the International Organization for Standardization [19]. To calculate intraobserver repeatability, the following parameters were obtained from three repeated measurements performed by each observer: the within-subject standard deviation (Sw) [21]; the precision (*p* = 1.96 × Sw), which defines the difference between the measurement in a patient with the true value for 95% of observations [21]; the repeatability limit (r = 2.77 × Sw), which can be interpreted as the difference between two measurements of the same patient for 95% of pairs of observation [21]; the coefficient of variation (CV); percentage value of the variation in the measurement and defined as the ratio of the Sw to the overall mean [CV = Sw/mean × 100 (%)]) [21]; and the intraclass correlation coefficient (ICC; classified as follows: less than 0.75 = poor agreement; 0.75 to less than 0.90 = moderate agreement; 0.90 or greater = high agreement) [22]. Analysis of variance (ANOVA) for repeated measurements was used to compare the three measurements with the MYAH and Myopia Master for all study variables (AL, K1, K2, and WTW). The estimated sample size calculation with a confidence level of 15% for 3 repeated measures was 42 subjects [23].

The agreement assessments between the MYAH and Myopia Master measurements (interdevice agreement) and between the observers (interobserver agreement) were analysed using the ICC, the Student paired *t* test (*p* values less than 0.05 were considered statistically significant) and the Bland–Altman method [24]. The 95% LoAs were calculated as the mean difference of ±1.96 SD [24]. The upper and lower limits of the 95% confidence intervals of the 95% LoAs were also calculated [25]. Linear regression was used to quantify the R^2^ correlation coefficient between measurements of both devices and both observers (*p* values less than 0.05 were considered statistically significant).

## 3. Results

### 3.1. Patient Demographics

A total of 42 right eyes of 42 patients (26 females and 16 males) with a mean age of 24.7 ± 5.80 years (range 19 to 38 years) and a mean spherical equivalent refractive error of −2.18 ± 1.88 D (range −0.25 to −6.00 D) were included in this study.

### 3.2. Intraobserver Repeatability

Table 1 shows the intraobserver repeatability results for AL, K1, K2, and WTW distance measured by the expert and non-expert observer with MYAH and Myopia Master devices. No statistically significant differences between the consecutive measurements for any parameter with any of the devices (*p* > 0.05 ANOVA for repeated measurements) were found with any device. Good ICCs (higher than 0.98) were found for all the analysed parameters, which indicates excellent repeatability for measurements collected by both the expert and non-expert observer with both devices. The intraobserver repeatability was practically identical in all parameters between the expert and non-expert with both the MYAH and the Myopia Master devices. AL measurements showed the best repeatability results with the MYAH and Myopia Master (ICC = 1.0; CV ≤ 0.06% for both observers), while the WTW distance presented poorer results (ICC ≤ 0.991; CV ≤ 0.52%).

### 3.3. Interdevice Agreement (MYAH vs. Myopia Master)

**Figure 1:**
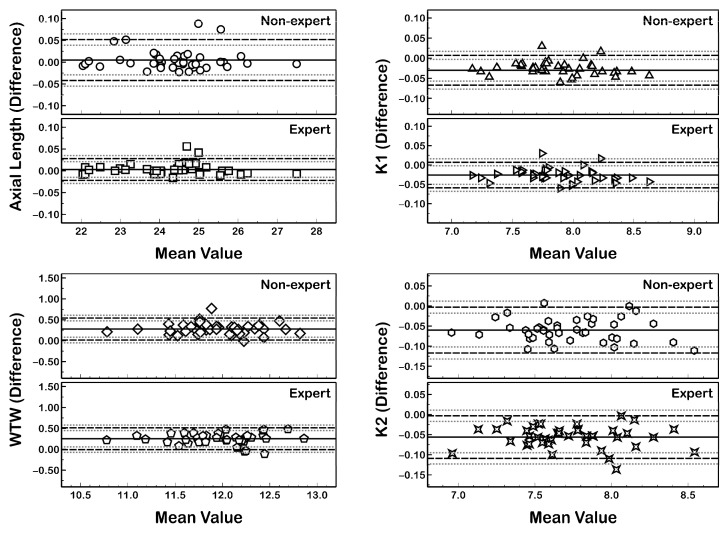
Bland–Altman plots of the agreement in AL, K1 (flat keratometry), K2 (steep keratometry), and WTW (white-to-white) distance measurements between the MYAH and Myopia Master collected by the non-expert and expert observer. The mean difference (solid line), 95% limit of agreement (LoA) (black discontinuous lines), and 95% confidence interval of LoA (grey discontinuous lines) are represented.

The agreement of the measurements between the MYAH and the Myopia Master for each of the observers is summarized in Table 2 and Figure 1.

**Figure 2:**
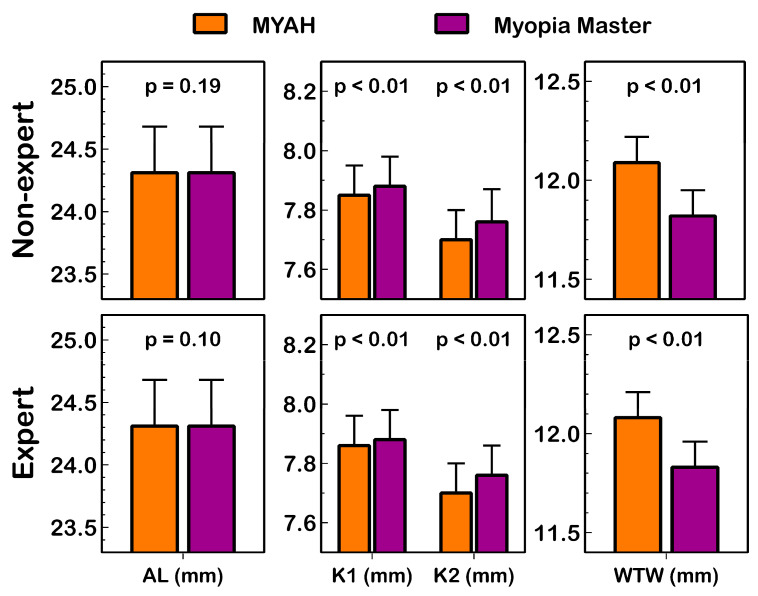
Summary of AL (axial length), K1 (flat keratometry), K2 (steep keratometry), and WTW (white-to-white) distance measured with MYAH and Myopia Master by both observers. The paired *t* test *p* value and 95% confidence interval are presented.

Statistically significant differences (*p* ≤ 0.01 paired *t* test) in the K1, K2, and WTW distances between both devices were found in measurements collected by both the expert and the non-expert observer (Table 2; Figure 2). In contrast, the AL measurement showed a non-statistically significant difference (*p* ≥ 0.10) between the MYAH and Myopia Master in both the expert and non-expert observer.

**Figure 3:**
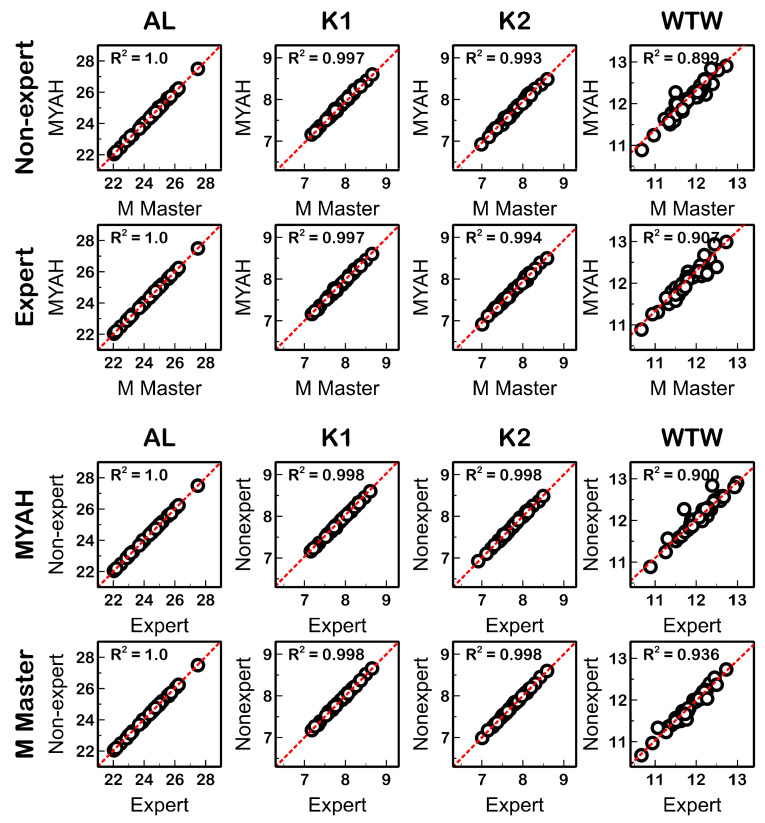
Correlations of AL (axial length), K1 (flat keratometry), K2 (steep keratometry), and WTW (white-to-white) distance measured with MYAH and Myopia Master by both observers (**top**) and by expert and non-expert with MYAH and Myopia Master (**bottom**). The red dotted lines represent the trend line of the correlations.

The Myopia Master significantly underestimated (*p* ≤ 0.01) K1 and K2 (providing flatter or lower corneal power) and WTW distance values compared with the MYAH (Figure 2). An excellent correlation of all ocular parameters was found between both devices (R^2^ ≥ 0.899; *p* < 0.01; Figure 3).

### 3.4. Interobserver Agreement (between Expert and Non-Expert)

**Figure 4:**
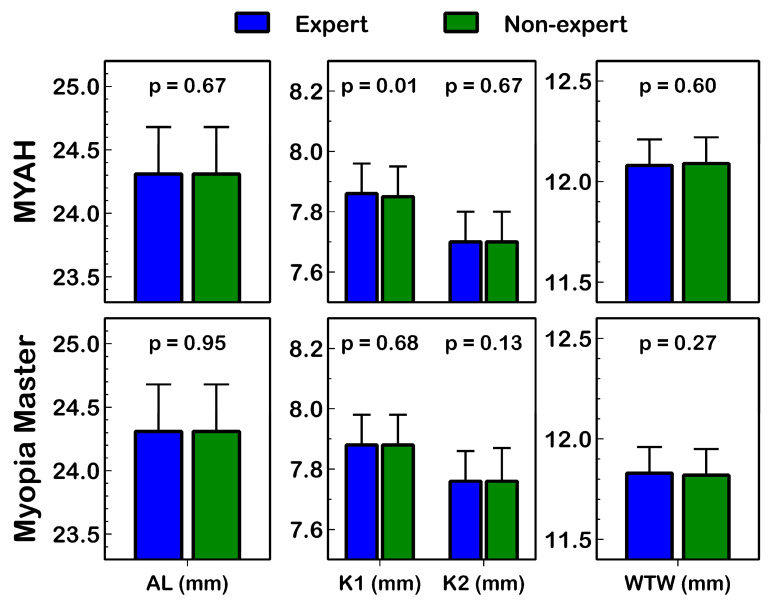
Summary of AL (axial length), K1 (flat keratometry), K2 (steep keratometry), and WTW (white-to-white) distance measured by expert and non-expert observers with MYAH and Myopia Master. The paired *t* test *p* value and 95% confidence interval are presented.

Table 3 presents the agreement of the measurements collected by the expert and non-expert observer for each device and eye parameter. The level of experience of the observers did not seem to have an influence on the measurements because AL, K1, K2, and WTW parameters did not show statistically significant differences (*p* ≥ 0.13; Figure 4) between the expert and the non-expert observer measurements with both devices. In addition, a high correlation between the measurements of the expert and the non-expert observer was found for all variables (R^2^ ≥ 0.90; *p* ≤ 0.01; Figure 3).

## 4. Discussion

In the field of myopia control management, repeatable and accurate biometric measurements are of paramount relevance for monitoring eye AL, especially in the paediatric population [26]. Therefore, new biometric devices, such as the MYAH and Myopia Master devices, have been developed and launched on the market due to the increase in myopic patients in the general population and the interest of eye care practitioners in reducing myopia progression.

Therefore, this study analysed the repeatability and agreement of MYAH and Myopia Master outcomes in a sample of healthy subjects to provide a clinical validation of these measurements, which is of interest to a wide number of eye care practitioners who provide myopia management treatments, with a special analysis of the impact on biometric outcomes related with the experience of the observers. It is common for repeatability and accuracy studies that compare different devices to be carried out by experienced observers who perform the measurements [13,14], but little information is provided about the impact of the experience of the observers on the quality of the eye parameter measurements.

Therefore, the main novelty of this study is that measurements of two different observers with large differences in clinical practice experience (an expert versus a non-expert final-year optometry degree student) were assessed and compared to determine whether the level of experience of the observer influences the repeatability and accuracy of the biometric measurements provided by these new devices. This was performed as the acquisition procedure with both devices requires that operators centre the image on the display following the alignment guides in view of both devices do not include a self-centering tool that automatises the capture procedure. These results are of interest to clinical practioners in the management of myopia control as this study demonstrates that any practitioner can obtain reliable measurements with these devices, regardless of the number of devices the clinician has previously handled.

The intraobserver repeatability of AL, K1, K2, and WTW measurements performed with the MYAH and Myopia Master device showed a very good ICC (ICC ≥ 0.981) and CV (CV ≤ 0.52%) for both the expert and non-expert observer. Therefore, the clinical practice experience of the observers has no significant impact on the repeatability of biometric parameters measured with both devices (Figure 1).

Better repeatability values found correspond with the AL measurements (Sw of 0.01 mm and ICC of 1.0) with both devices and both observers. This result agrees with a recent publication of the repeatability of the MYAH device in the paediatric population [13] with a Sw of 0.02 mm and an ICC of 1.0. On the other hand, to the best of the knowledge of the authors, only one report has described the repeatability of the Myopia Master [14] in a population of children with myopia, with the same ICC value (ICC = 1.0) [13].

The IOL Master 700 biometer (Carl Zeiss Meditec AG, Jena, Germany) is currently considered the gold standard in AL measurement [27]. Garza-Leon et al. [28] studied the repeatability of AL measurements provided by the IOL Master 700 in a sample of 45 adult subjects and obtained a Sw of 0.0079 mm and an ICC of 1.0. Another of the most widely used biometers in clinical practice is the Lenstar LS 900 (Haag-Streit AG, Koeniz, Switzerland), which also has very good repeatability in the measurement of AL in adults (Sw of 0.018 mm) [29]. From these results, it can be deduced that the MYAH and Myopia Master devices provide good repeatability of AL measurements, comparable to that previously reported for the IOL Master 700 [28] or Lenstar LS 900 [29].

Regarding the MYAH device’s corneal curvature measurements, the repeatability of K1 and K2 measurements collected by the expert observer (Sw of 0.01; CV of 0.14 and 0.19%, respectively) was slightly better than the repeatability achieved by the non-expert observer (Sw of 0.02; CV of 0.20% for both K1 and K2 measurements) but very similar to previously reported [13] repeatability values in a paediatric population (Sw of 0.02; and CV of 0.22% and 0.28%, respectively). The same trend was found with the Myopia Master corneal curvature measurements, with repeatability for K1 and K2 measurements of Sw of 0.01 and CV of 0.12% and 0.19%, respectively, for expert measurements, and Sw of 0.01 and 0.02 and CV of 0.12% and 0.19%, respectively, for non-expert observer measurements. This is the first evidence of repeatability values for K1 and K2 measurements provided by the Myopia Master in the literature.

Although the corneal diameter or WTW distance measurements are not relevant parameters in the control of myopia, they are important parameters in contact lens fitting, such as orthokeratology [6] and other eye-care procedures. The WTW measurement shows good repeatability (ICC ≥ 0.98) with both the MYAH and the Myopia Master, although this parameter obtained the worst repeatability compared with AL, K1, or K2 measurements. These findings agree with previous reports [12] that found close repeatability for WTW measurements collected with the MYAH in children (Sw of 0.07 and ICC of 0.97) [13] and collected with the Myopia Master [14] in myopic spectacle wearers (ICC of 0.96) and orthokeratology patients (ICC of 0.98). Additionally, WTW repeatability is comparable with results achieved in the adult population with other devices, such as the IOL Master 700 (Sw of 0.05 mm; ICC of 0.990) [30], Galilei G2 (Sw of 0.03 mm; ICC of 0.995) [30], or DRI OCT Triton (Sw of 0.06 mm; ICC of 0.993) [30].

This study also observed excellent agreement between the expert and non-expert observer measurements for all assessed biometric parameters (AL, K1, K2, and WTW) with both devices, suggesting that the level of clinical experience of the eye care practitioner could be irrelevant in terms of myopia management with the MYAH or Myopia Master devices (Figure 4).

Moreover, the agreement between the MYAH and Myopia Master outcomes was assessed in this study. AL measurement is a fundamental parameter for monitoring myopia progression, and excellent (ICC = 1.0) agreement and non-statistically significant differences (*p* ≥ 0.10) between both devices were found, suggesting that the AL measurement of both devices could be interchangeable in the management of patients with myopia as well as follow-ups (Figure 2). Martinez-Plaza et al. [13] reported a similar agreement in AL measurements between the MYAH and Myopia Master, but, contrary to the results found in this study, their findings indicated statistically significant differences between AL measurements (*p* < 0.01) when collecting just one AL measurement with the Myopia Master device. These discrepancies may be related to differences in the study population explored because measurements in children [13] could be affected by patient co-operation, and further research in paediatric patients is necessary.

However, the MYAH corneal K1 and K2 measurements tended to be smaller (higher corneal power) and have a larger corneal diameter than those achieved with the Myopia Master device. Differences between the MYAH and Myopia Master for K1, K2, and WTW presented statistically significant differences but were small in magnitude. The MYAH device allows post-analysis manual adjustment of WTW distance; however, in this study, no adjustment was completed. Further research assessing the agreement between assessed devices with other devices that provide WTW distance is necessary to clarify if manual adjustment is required. Hence, the clinical relevance of these differences is debatable [31], and these measurements should be interpreted with caution in clinical practice.

Other studies have compared the agreement of the biometric measurements of the IOL Master 700 or Pentacam AXL with the MYAH [12,32] or Myopia Master [14,32,33,34]. No statistically significant differences were reported between AL measurements of the IOL Master and Pentacam AXL with the MYAH measurements (*p* = 0.06) [12]. However, statistically significant differences were reported regarding K1, K2, and WTW measurements between these three devices (*p* ≤ 0.01) [12]. A similar trend was described comparing IOL Master 700 and Myopia Master measurements in an adult cohort [33], without statistically significant differences in AL measurements but statistically significant differences in the remaining biometric parameter (K1, K2, or WTW) measurements. In contrast, a significantly shorter AL, flatter corneal curvatures, and smaller WTW outcomes were reported with Myopia Master compared to the IOL Master 700 in a paediatric population [14]. Mattern et al. [32] found no statistically significant difference in the mean-K measurement between the IOL Master 700 and the Myopia Master or MYAH in a sample of 22 children. Another study, published by Hessler et al. [34], also reported no statistically significant nor clinically relevant differences in the measurement of AL or mean-K between the IOL Master 700 and the Myopia Master. However, Garcia-Ardoy [14] found differences in AL and mean-K measurements between the IOL Master 700 and the Myopia Master in children who wore spectacle and orthokeratology contact lens.

The measurement of other parameters provided by the Myopia Master and the MYAH has also been compared to other devices [35]. For example, statistically significant differences have been reported in refractive measurements (spherical equivalent and cylindrical refraction) between the Myopia Master and the ARK-1 autorefractometer [35].

The main limitation of this study concerns the assessed population, as young adult subjects were included and could have shown better co-operation during measurements than paediatric children with myopia, who are the target population in myopia progression control. It is expected that patient co-operation during measurements could have some impact on the repeatability and accuracy of biometric measurements; consequently, this could be of interest for more research in the paediatric population. Additionally, it would be of interest to compare more devices, such as the IOL Master 700 (currently AL gold standard [27]) and others, and to assess the impact of different degrees of observer experience on their measurements. Moreover, a sample of healthy patients who are not undergoing any specific myopia control treatment, e.g., orthokeratology, were included, so it would be interesting to assess whether treatment via myopia control could affect the repeatability and accuracy of AL measurements. Finally, the expert observer had no experience with the MYAH or Myopia Master but has experience with other similar devices (e.g., IOL Master 500, IOL Master 700, Pentacam, etc.), which may limit the results of a real comparison between an experienced user and a novice observer in device use.

In summary, although it might be expected that the outcome of the devices would be affected by the level of professional experience of the eye care practitioner performing the measurements, the results of this study showed that for the MYAH and Myopia Master devices, this does not occur.

## 5. Conclusions

The MYAH and Myopia Master are highly repeatable devices that provide consistent measurements of the AL, K1, K2, and WTW distance in a healthy adult population regardless of the previous clinical practice experience of the observer who performs the measurements. The agreement between both instruments was excellent for AL measurements by both the expert and the non-expert observer, which indicates that the measurements could be used interchangeably. On the other hand, the measurements of corneal curvature and WTW distance provided by both devices could be used interchangeably with caution in clinical practice (though not in research) because both devices provide significantly different outcomes.

## Figures and Tables

**Table 1:** Intraobserver repeatability for all parameter measurements obtained with the MYAH and Myopia Master devices for each observer.

		Mean ± SD	Sw	P	r	CV (%)	ICC (95% CI)	*p* Value *
**MYAH (TOPCON)**								
Non-expert observer	AL (mm)	24.31 ± 1.19	0.01	0.02	0.03	0.05	1.00 (1.00–1.00)	0.52
	K1 (mm)	7.85 ± 0.33	0.02	0.03	0.04	0.20	0.999 (0.998–0.999)	0.70
	K2 (mm)	7.70 ± 0.33	0.02	0.03	0.04	0.20	0.999 (0.998–0.999)	0.32
	WTW (mm)	12.09 ± 0.41	0.06	0.12	0.18	0.52	0.981 (0.968–0.989)	0.93
Expert observer	AL (mm)	24.31 ± 1.19	0.01	0.02	0.03	0.05	1.00 (1.00–1.00)	0.11
	K1 (mm)	7.86 ± 0.33	0.01	0.02	0.03	0.14	0.999 (0.999–1.00)	0.54
	K2 (mm)	7.70 ± 0.33	0.01	0.03	0.04	0.19	0.999 (0.999–0.999)	0.87
	WTW (mm)	12.08 ± 0.43	0.06	0.12	0.16	0.49	0.987 (0.978–0.992)	0.67
**MYOPIA MASTER (OCULUS)**								
Non-expert observer	AL (mm)	24.31 ± 1.19	0.01	0.03	0.04	0.06	1.00 (1.00–1.00)	0.94
	K1 (mm)	7.88 ± 0.33	0.01	0.02	0.03	0.12	1.00 (0.999–1.00)	0.22
	K2 (mm)	7.76 ± 0.34	0.02	0.03	0.04	0.19	0.999 (0.998–0.999)	0.19
	WTW (mm)	11.82 ± 0.42	0.05	0.11	0.15	0.46	0.984 (0.973–0.991)	0.18
Expert observer	AL (mm)	24.31 ± 1.19	0.01	0.03	0.04	0.06	1.00 (1.00–1.00)	0.83
	K1 (mm)	7.88 ± 0.33	0.01	0.02	0.03	0.12	1.00 (0.999–1.00)	0.23
	K2 (mm)	7.76 ± 0.34	0.01	0.03	0.04	0.19	0.999 (0.998–0.999)	0.18
	WTW (mm)	11.83 ± 0.43	0.05	0.10	0.14	0.43	0.991 (0.985–0.995)	0.20

AL = axial length; K1 = flat keratometry; K2 = steep keratometry; WTW = white-to-white distance; SD = standard deviation; Sw = within-subject standard deviation; P= precision; r = limit of repeatability; CV = coefficient of variation; ICC = intraclass correlation coefficient; CI = confidence interval. * ANOVA for repeated measurements (*p* ≤ 0.05 was considered statistically significant).

**Table 2:** Interdevice agreement for all parameter measurements obtained with the MYAH and Myopia Master devices for each observer.

		Diff Mean ± SD	LoA 95%	ICC (95% CI)	*p* Value *
**NON-EXPERT OBSERVER**					
MYAH- Myopia Master	AL (mm)	0.00 ± 0.02	−0.04 to 0.05	1.00 (1.00–1.00)	0.19
	K1 (mm)	−0.03 ± 0.02	−0.07 to 0.01	0.999 (0.998–1.00)	<0.01
	K2 (mm)	−0.06 ± 0.03	−0.12 to 0.00	0.998 (0.997–0.999)	<0.01
	WTW (mm)	0.28 ± 0.13	0.02 to 0.54	0.974 (0.951–0.986)	<0.01
**EXPERT OBSERVER**					
MYAH- Myopia Master	AL (mm)	0.00 ± 0.01	−0.02 to 0.03	1.00 (1.00–1.00)	0.10
	K1 (mm)	−0.03 ± 0.02	−0.06 to 0.01	0.999 (0.999–1.00)	<0.01
	K2 (mm)	−0.06 ± 0.03	−0.11 to 0.00	0.998 (0.997–0.999)	<0.01
	WTW (mm)	0.25 ± 0.13	−0.01 to 0.51	0.975 (0.954–0.987)	<0.01

AL = axial length; K1 = flat keratometry; K2 = steep keratometry; WTW = white-to-white distance; SD = standard deviation; LoA = limit of agreement; ICC = intraclass correlation coefficient; CI = confidence interval. * Paired *t* test (*p* ≤ 0.05 was considered statistically significant).

**Table 3:** Interobserver agreement for all parameters between the measures taken by each observer obtained with the MYAH and Myopia Master.

		Diff Mean ± SD	LoA 95%	ICC (95% CI)	*p* Value *
**MYAH (TOPCON)**					
Non-expert vs. Expert	AL (mm)	0.00 ± 0.02	−0.02 to 0.05	1.00 (1.00–1.00)	0.67
	K1 (mm)	−0.01 ± 0.01	−0.03 to 0.02	1.00 (0.999–1.00)	0.12
	K2 (mm)	0.00 ± 0.02	−0.03 to 0.03	0.999 (0.999–1.00)	0.67
	WTW (mm)	0.01 ± 0.14	−0.26 to 0.28	0.973 (0.950–0.985)	0.60
**MYOPIA MASTER (OCULUS)**					
Non-expert vs. Expert	AL (mm)	0.00 ± 0.02	−0.05 to 0.05	1.00 (1.00–1.00)	0.95
	K1 (mm)	0.00 ± 0.01	−0.03 to 0.03	1.00 (0.999–1.00)	0.68
	K2 (mm)	0.00 ± 0.01	−0.02 to 0.03	1.00 (0.999–1.00)	0.13
	WTW (mm)	−0.01 ± 0.08	−0.18 to 0.15	0.991 (0.982–0.995)	0.27

AL = axial length; K1 = flat keratometry; K2 = steep keratometry; WTW = white-to-white distance; SD = standard deviation; LoA = limit of agreement; ICC = intraclass correlation coefficient; CI = confidence interval. * Paired *t* test (*p* ≤ 0.05 was considered statistically significant).

## Data Availability

The data presented in this study are available upon reasonable request from the corresponding author.
